# Supplementary material for: Vibronic effects on the quantum tunnelling of magnetisation in Kramers single-molecule magnets
Source: Nat Commun. 2024 Jan 12;15:485. doi: 10.1038/s41467-023-44486-3 (PMC10784566; doi:10.1038/s41467-023-44486-3)
Supplement: Supplementary file 1 — Supplementary Information [file 41467_2023_44486_MOESM1_ESM.pdf]

Supplementary Information:

Vibronic Effects on the Quantum Tunnelling of Magnetisation in Kramers Single-Molecule Magnets

Andrea Mattioni,[1,*] Jakob K. Staab,[1] William J. A. Blackmore,[1] Daniel Reta,[1,2,3,4]
Jake Iles-Smith,[5] Ahsan Nazir,[5] and Nicholas F. Chilton[1,†]

[1]*Department of Chemistry, School of Natural Sciences, The University of Manchester, Oxford Road, Manchester, M13 9PL, UK*
[2]*Faculty of Chemistry, The University of the Basque Country UPV/EHU, Donostia, 20018, Spain*
[3]*Donostia International Physics Center (DIPC), Donostia, 20018, Spain*
[4]*IKERBASQUE, Basque Foundation for Science, Bilbao, 48013, Spain*
[5]*Department of Physics and Astronomy, School of Natural Sciences, The University of Manchester, Oxford Road, Manchester M13 9PL, UK*

CONTENTS

Supplementary Note 1. Spin-phonon couplings and phonon density of states 2

Supplementary Note 2. Derivation of the effective vibronic doublet Hamiltonian 3
A. Electronic perturbation theory 3
B. Polaron Hamiltonian for the ground doublet 4
C. Landau-Zener probability 6

Supplementary Note 3. Distribution of spin-phonon coupling vectors 8

Supplementary Note 4. Ground Zeeman splitting for [Dy(bbpen)Br] 9

Supplementary Note 5. Estimate of the internal fields 10
A. Dipolar fields 10
B. Hyperfine coupling 11

Supplementary Note 6. Relation between single-mode axiality and spin-flip probability 13

Supplementary references 15

* andrea.mattioni@manchester.ac.uk
† nicholas.chilton@manchester.ac.uk

Supplementary Note 1. SPIN-PHONON COUPLINGS AND PHONON DENSITY OF STATES

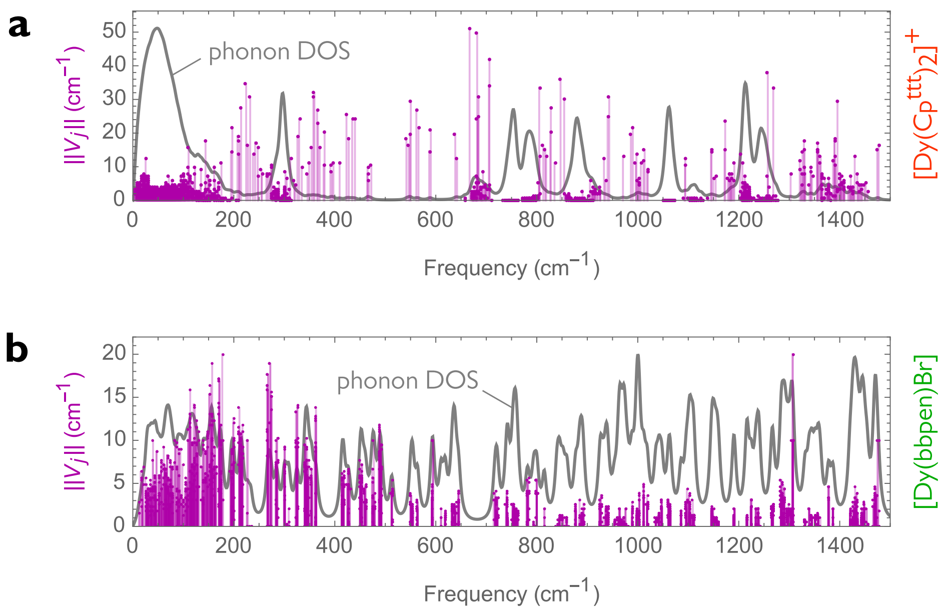

Supplementary Figure 1. **Spin-phonon couplings and phonon density of states.** Spin phonon-couplings (purple sticks) are quantified as the Frobenius norm of the electronic part of the spin-phonon coupling operators $\hat{V}_j$. The phonon density of states (DOS), shown as a grey line, is obtained by broadening each mode with an anti-symmetrised Lorentzian lineshape with full width at half maximum of 10 cm^{-1} [1]. **a**, $[Dy(Cp^{ttt})_2]^+$ in dichloromethane solvent ball; **b**, [Dy(bbpen)Br] molecular crystal.

Supplementary Note 2. DERIVATION OF THE EFFECTIVE VIBRONIC DOUBLET HAMILTONIAN

A. Electronic perturbation theory

The starting point for our analysis of vibronic effects on QTM is the vibronic Hamiltonian

$$\hat{H} = \sum_{m>0} E_m(|m\rangle\langle m| + |\bar{m}\rangle\langle\bar{m}|) + \hat{H}_{\text{Zee}} + \sum_j \hat{V}_j \otimes (\hat{b}_j + \hat{b}_j^\dagger) + \sum_j \omega_j \hat{b}_j^\dagger \hat{b}_j, \tag{S1}$$

where $\hat{H}_{\text{Zee}} = \mu_B g_J \mathbf{B}\cdot\hat{\mathbf{J}}$. is the Zeeman interaction with a magnetic field $\mathbf{B}$. The doubly degenerate eigenstates of the crystal field Hamiltonian $H_{\text{CF}} = \sum_{m>0} E_m(|m\rangle\langle m| + |\bar{m}\rangle\langle\bar{m}|)$ are related by time-reversal symmetry, i.e. $\hat{\Theta}|m\rangle \propto |\bar{m}\rangle$ with $\hat{\Theta}^2|m\rangle = -|m\rangle$, where $\hat{\Theta}$ is the time-reversal operator. In the case of Dy(III), the total electronic angular momentum is $J = 15/2$, leading to $2J+1 = 16$ electronic states. We label these states in ascending energy with integers $m = \pm 1, \ldots, \pm 8$, using the compact notation $|-m\rangle = |\bar{m}\rangle$.

We momentarily neglect the spin-phonon coupling and focus on the purely electronic Hamiltonian $H_{\text{el}} = H_{\text{CF}} + H_{\text{Zee}}$. Within each degenerate subspace, the Zeeman term selects a specific electronic basis and lifts its degeneracy. This can be seen by projecting the electronic Hamitonian onto the m-th subspace and diagonalising the 2×2 matrix

$$H_{\text{el}}^{(m)} = E_m + \mu_{\text{B}} g_J \begin{pmatrix} \langle m|\mathbf{B}\cdot\hat{\mathbf{J}}|m\rangle & \langle m|\mathbf{B}\cdot\hat{\mathbf{J}}|\bar{m}\rangle \\ \langle\bar{m}|\mathbf{B}\cdot\hat{\mathbf{J}}|m\rangle & \langle\bar{m}|\mathbf{B}\cdot\hat{\mathbf{J}}|\bar{m}\rangle \end{pmatrix}. \tag{S2}$$

For each individual cartesian component of the angular momentum, we decompose the corresponding 2×2 matrix in terms of Pauli spin operators, which allows to rewrite the Hamiltonian of the m-th doublet as $H_{\text{el}}^{(m)} = E_m + \mu_{\text{B}}\mathbf{B}\cdot\mathbf{g}_{\text{el}}^{(m)}\cdot\boldsymbol{\sigma}^{(m)}/2$, where

$$\mathbf{g}_{\text{el}}^{(m)} = 2g_J \begin{pmatrix} \Re\langle\bar{m}|\hat{J}_x|m\rangle & \Im\langle\bar{m}|\hat{J}_x|m\rangle & \langle m|\hat{J}_x|m\rangle \\ \Re\langle\bar{m}|\hat{J}_y|m\rangle & \Im\langle\bar{m}|\hat{J}_y|m\rangle & \langle m|\hat{J}_y|m\rangle \\ \Re\langle\bar{m}|\hat{J}_z|m\rangle & \Im\langle\bar{m}|\hat{J}_z|m\rangle & \langle m|\hat{J}_z|m\rangle \end{pmatrix} \tag{S3}$$

is the g-matrix for an effective spin 1/2 and $\boldsymbol{\sigma}^{(m)} = (\sigma_x^{(m)}, \sigma_y^{(m)}, \sigma_z^{(m)})$, with $\sigma_z^{(m)} = |m\rangle\langle m| - |\bar{m}\rangle\langle\bar{m}|$. We note that in general the g-matrix in Eq. (S3) is not hermitean, but can be brought to such form by transforming the spin operators $\boldsymbol{\sigma}^{(m)}$ to an appropriate basis [2]. An easier prescription to find the hermitean form af any g-matrix $\mathbf{g}$ is to redefine it as $\sqrt{\mathbf{g}\mathbf{g}^\dagger}$.

To lowest order in the magnetic field, the Zeeman interaction lifts the two-fold degeneracy by selecting the basis

$$|m_+\rangle = \cos\tfrac{\theta_m}{2}|m\rangle + e^{\mathrm{i}\phi_m}\sin\tfrac{\theta_m}{2}|\bar{m}\rangle \tag{S4}$$

$$|m_-\rangle = -\sin\tfrac{\theta_m}{2}|m\rangle + e^{\mathrm{i}\phi_m}\cos\tfrac{\theta_m}{2}|\bar{m}\rangle \tag{S5}$$

and shifting the energies according to $E_{m,\pm} = E_m \pm \Delta_m/2$, where the gap

$$\begin{aligned} \Delta_m &= \langle m_+|\hat{H}_{\text{Zee}}|m_+\rangle - \langle m_-|\hat{H}_{\text{Zee}}|m_-\rangle \\ &= 2\mu_{\text{B}} g_J \sqrt{\langle m|\mathbf{B}\cdot\hat{\mathbf{J}}|m\rangle^2 + |\langle m|\mathbf{B}\cdot\hat{\mathbf{J}}|\bar{m}\rangle|^2} \end{aligned} \tag{S6}$$

can be obtained as the norm of the vector $\mathbf{j}_m = \mu_{\text{B}}\mathbf{B}\cdot\mathbf{g}_{\text{el}}^{(m)}$ and the phase and mixing angles are defined as

$$e^{\mathrm{i}\phi_m} = \frac{\langle\bar{m}|\mathbf{B}\cdot\hat{\mathbf{J}}|m\rangle}{|\langle\bar{m}|\mathbf{B}\cdot\hat{\mathbf{J}}|m\rangle|}, \qquad \tan\theta_m = \frac{|\langle\bar{m}|\mathbf{B}\cdot\hat{\mathbf{J}}|m\rangle|}{\langle m|\mathbf{B}\cdot\hat{\mathbf{J}}|m\rangle}, \tag{S7}$$

or equivalently as the azimuthal and polar angles determining the direction of $\mathbf{j}_m$.

Besides selecting a preferred basis and lifting the degeneracy of each doublet, the Zeeman interaction also causes mixing between different doublets. In particular, the lowest doublet will change according to

$$|1'_\pm\rangle = |1_\pm\rangle + \sum_{m\neq 1,\bar{1}} |m\rangle \frac{\langle m|\hat{H}_{\text{Zee}}|1_\pm\rangle}{E_1 - E_m} + \mathcal{O}(B^2) \approx \left(1 - \hat{Q}_1\hat{H}_{\text{Zee}}\right)|1_\pm\rangle, \tag{S8}$$

with

$$\hat{Q}_1 = \sum_{m\neq 1,\bar{1}} |m\rangle \frac{1}{E_m - E_1}\langle m|. \tag{S9}$$

B. Polaron Hamiltonian for the ground doublet

Now that we have an approximate expression for the relevant electronic states, we reintroduce the spin-phonon coupling into the picture. First, we project the vibronic Hamiltonian (S1) onto the subspace spanned by $|1'_\pm\rangle$, yielding

$$\hat{H}_{\text{eff}} = E_1 + \begin{pmatrix} \frac{\Delta_1}{2} & 0 \\ 0 & -\frac{\Delta_1}{2} \end{pmatrix} + \sum_j \begin{pmatrix} \langle 1'_+|\hat{V}_j|1'_+\rangle & \langle 1'_+|\hat{V}_j|1'_-\rangle \\ \langle 1'_-|\hat{V}_j|1'_+\rangle & \langle 1'_-|\hat{V}_j|1'_-\rangle \end{pmatrix} \otimes (\hat{b}_j + \hat{b}_j^\dagger) + \sum_j \omega_j \hat{b}_j^\dagger \hat{b}_j. \tag{S10}$$

On this basis, the purely electronic part $\hat{H}_{\text{CF}} + \hat{H}_{\text{Zee}}$ is diagonal with eigenvalues $E_1 \pm \Delta_1/2$, and the purely vibrational part is trivially unaffected. On the other hand, the spin-phonon couplings can be calculated to lowest order in the magnetic field strength B as

$$\begin{aligned} \langle 1'_\pm|\hat{V}_j|1'_\pm\rangle &= \langle 1_\pm|\left(1-\hat{H}_{\text{Zee}}\hat{Q}_1\right)\hat{V}_j\left(1-\hat{Q}_1\hat{H}_{\text{Zee}}\right)|1_\pm\rangle + \mathscr{O}(B^2) \\ &= \langle 1_\pm|\hat{V}_j|1_\pm\rangle - \langle 1_\pm|\left(\hat{V}_j\hat{Q}_1\hat{H}_{\text{Zee}} + \hat{H}_{\text{Zee}}\hat{Q}_1\hat{V}_j\right)|1_\pm\rangle + \mathscr{O}(B^2) \\ &= \langle 1|\hat{V}_j|1\rangle - \langle 1_\pm|\hat{W}_j|1_\pm\rangle + \mathscr{O}(B^2), \end{aligned} \tag{S11}$$

$$\begin{aligned} \langle 1'_\mp|\hat{V}_j|1'_\pm\rangle &= \langle 1_\mp|\left(1-\hat{H}_{\text{Zee}}\hat{Q}_1\right)\hat{V}_j\left(1-\hat{Q}_1\hat{H}_{\text{Zee}}\right)|1_\pm\rangle + \mathscr{O}(B^2) \\ &= \langle 1_\mp|\hat{V}_j|1_\pm\rangle - \langle 1_\mp|\left(\hat{V}_j\hat{Q}_1\hat{H}_{\text{Zee}} + \hat{H}_{\text{Zee}}\hat{Q}_1\hat{V}_j\right)|1_\pm\rangle + \mathscr{O}(B^2) \\ &= -\langle 1_\mp|\hat{W}_j|1_\pm\rangle + \mathscr{O}(B^2), \end{aligned} \tag{S12}$$

where we have defined

$$\hat{W}_j = \hat{V}_j\hat{Q}_1\hat{H}_{\text{Zee}} + \hat{H}_{\text{Zee}}\hat{Q}_1\hat{V}_j \tag{S13}$$

and used the time-reversal invariance of the spin-phonon coupling operators to obtain $\langle 1_\pm|\hat{V}_j|1_\pm\rangle = \langle 1|\hat{V}_j|1\rangle$ and $\langle 1_\mp|\hat{V}_j|1_\pm\rangle = 0$.

The two states $|1_\pm\rangle$ form a conjugate pair under time reversal, meaning that $\hat{\Theta}|1_\pm\rangle = \mp e^{i\alpha}|1_\mp\rangle$ for some $\alpha \in \mathbb{R}$. Using the fact that for any two states ψ, φ, and for any operator $\hat{O}$ we have $\langle\psi|\hat{O}|\varphi\rangle = \langle\hat{\Theta}\varphi|\hat{\Theta}\hat{O}^\dagger\hat{\Theta}^{-1}|\hat{\Theta}\psi\rangle$, and recalling that the angular momentum operator is odd under time reversal, i.e. $\hat{\Theta}\hat{\mathbf{J}}\hat{\Theta}^{-1} = -\hat{\mathbf{J}}$, we can show that

$$\langle 1_-|\hat{W}_j|1_-\rangle = \langle\hat{\Theta}1_-|\hat{\Theta}\hat{W}_j\hat{\Theta}^{-1}|\hat{\Theta}1_-\rangle = -\langle 1_+|\hat{W}_j|1_+\rangle.$$

Keeping in mind these observations, and defining the vector

$$\mathbf{w}_j = \begin{pmatrix} w_j^x \\ w_j^y \\ w_j^z \end{pmatrix} = \begin{pmatrix} \Re\,\langle 1_-|\hat{W}_j|1_+\rangle \\ \Im\,\langle 1_-|\hat{W}_j|1_+\rangle \\ \langle 1_+|\hat{W}_j|1_+\rangle \end{pmatrix}, \tag{S14}$$

we can rewrite the spin-phonon coupling operators in Eq. (S10) as

$$\begin{pmatrix} \langle 1'_+|\hat{V}_j|1'_+\rangle & \langle 1'_+|\hat{V}_j|1'_-\rangle \\ \langle 1'_-|\hat{V}_j|1'_+\rangle & \langle 1'_-|\hat{V}_j|1'_-\rangle \end{pmatrix} = \langle 1|\hat{V}_j|1\rangle - \begin{pmatrix} \langle 1_+|\hat{W}_j|1_+\rangle & \langle 1_-|\hat{W}_j|1_+\rangle^* \\ \langle 1_-|\hat{W}_j|1_+\rangle & -\langle 1_+|\hat{W}_j|1_+\rangle \end{pmatrix} = \langle 1|\hat{V}_j|1\rangle - \mathbf{w}_j\cdot\boldsymbol{\sigma}' \tag{S15}$$

where $\boldsymbol{\sigma}'$ is a vector whose entries are the Pauli matrices in the basis $|1'_\pm\rangle$, i.e. $\sigma'_z = |1'_+\rangle\langle 1'_+| - |1'_-\rangle\langle 1'_-|$. Plugging this back into Eq. (S10) and explicitly singling out the diagonal components of $\hat{H}_{\text{eff}}$ in the basis $|1'_\pm\rangle$, we obtain

$$\begin{aligned} \hat{H}_{\text{eff}} &= |1'_+\rangle\langle 1'_+|\left[E_1 + \frac{\Delta_1}{2} + \sum_j\left(\langle 1|\hat{V}_j|1\rangle - w_j^z\right)\left(\hat{b}_j + \hat{b}_j^\dagger\right) + \sum_j \omega_j\hat{b}_j^\dagger\hat{b}_j\right] \\ &+ |1'_-\rangle\langle 1'_-|\left[E_1 - \frac{\Delta_1}{2} + \sum_j\left(\langle 1|\hat{V}_j|1\rangle + w_j^z\right)\left(\hat{b}_j + \hat{b}_j^\dagger\right) + \sum_j \omega_j\hat{b}_j^\dagger\hat{b}_j\right] \\ &- \sum_j\left(w_j^x\sigma'_x + w_j^y\sigma'_y\right)\left(\hat{b}_j + \hat{b}_j^\dagger\right). \end{aligned} \tag{S16}$$

At this point, we apply a unitary polaron transformation to the Hamiltonian (S16)

$$\begin{aligned} \hat{S} &= \exp\left[\sum_{s=\pm}|1'_s\rangle\langle 1'_s|\sum_j\frac{1}{\omega_j}\left(\langle 1|\hat{V}_j|1\rangle - s w_j^z\right)\left(\hat{b}_j^\dagger - \hat{b}_j\right)\right] \\ &= \sum_{s=\pm}|1'_s\rangle\langle 1'_s|\prod_j\hat{D}_j(\xi_j^s) \end{aligned} \tag{S17}$$

where $\xi_j^s = \left(\langle 1|\hat{V}_j|1\rangle - s w_j^z\right)/\omega_j$ and

$$\hat{D}_j(\xi_j^s) = e^{\xi_j^s\left(\hat{b}_j^\dagger - \hat{b}_j\right)} \tag{S18}$$

is the bosonic displacement operator acting on mode j, i.e. $\hat{D}_j(\xi)\hat{b}_j\hat{D}_j^\dagger(\xi) = \hat{b}_j - \xi$. The Hamiltonian thus becomes

$$\hat{S}\hat{H}_{\text{eff}}\hat{S}^\dagger = \sum_{s=\pm} |1'_s\rangle\langle 1'_s| \left(E_1 + s\frac{\Delta_1}{2} - \sum_j \omega_j|\xi_j^s|^2\right) + \sum_j \omega_j \hat{b}_j^\dagger\hat{b}_j - \sum_j \hat{S}\left(w_j^x\sigma'_x + w_j^y\sigma'_y\right)\left(\hat{b}_j + \hat{b}_j^\dagger\right)\hat{S}^\dagger. \tag{S19}$$

The polaron transformation reabsorbes the diagonal component of the spin-phonon coupling (S15) proportional to w_j^z into the energy shifts $\omega_j|\xi_j^\pm|^2$, leaving a residual off-diagonal spin-phonon coupling proportional to w_j^x and w_j^y. Note that the polaron transformation exactly diagonalises the Hamiltonian (S10) if $w_j^x = w_j^y = 0$. In Supplementary Note 3, we argue in detail that in our case $|w_j^x|, |w_j^y| \ll |w_j^z|$ to a very good approximation. Based on this argument, we could decide to neglect the residual spin-phonon coupling in the polaron frame. The energies of the states belonging to the lowest doublet are shifted by a vibronic correction

$$E_{1'_\pm} = E_1 \pm \frac{\Delta_1}{2} - \sum_j \frac{1}{\omega_j}\left(\langle 1|\hat{V}_j|1\rangle \mp w_j^z\right)^2 \tag{S20}$$

$$= E_1 \pm \frac{\Delta_1}{2} - \sum_j \frac{1}{\omega_j}\left(\langle 1|\hat{V}_j|1\rangle^2 \mp 2\langle 1|\hat{V}_j|1\rangle w_j^z + \mathscr{O}(B^2)\right), \tag{S21}$$

leading to a redefinition of the energy gap

$$E_{1'_+} - E_{1'_-} = \Delta_1 + 4\sum_j \frac{\langle 1|\hat{V}_j|1\rangle}{\omega_j} w_j^z. \tag{S22}$$

Although the off-diagonal components of the spin-phonon coupling w_j^x and w_j^y are several orders of magnitude smaller than the diagonal one w_j^z (see Supplementary Note 3), the sheer number of vibrational modes could still lead to an observable effect on the electronic degrees of freedom. We can estimate this effect by averaging the residual spin-phonon coupling over a thermal phonon distribution in the polaron frame. Making use of Eq. (S17), the off-diagonal coupling in Eq. (S19) can be written as

$$\hat{H}_{\text{sp-ph}}^{(\text{pol})} = -\sum_j \hat{S}\left(w_j^x\sigma'_x + w_j^y\sigma'_y\right)\left(\hat{b}_j + \hat{b}_j^\dagger\right)\hat{S}^\dagger \tag{S23}$$

$$= -\sum_j |1'_-\rangle\langle 1_-|\hat{W}_j|1_+\rangle\langle 1'_+|\, \hat{D}_j(\xi_j^-)\left(\hat{b}_j + \hat{b}_j^\dagger\right)\hat{D}_j^\dagger(\xi_j^+) + \text{h.c.}$$

Assuming the vibrations to be in a thermal state at temperature T in the polaron frame

$$\rho_{\text{ph}}^{(\text{th})} = \prod_j \rho_j^{(\text{th})} = \prod_j \frac{e^{-\omega_j\hat{b}_j^\dagger\hat{b}_j/k_{\text{B}}T}}{\text{Tr}\left[e^{-\omega_j\hat{b}_j^\dagger\hat{b}_j/k_{\text{B}}T}\right]}, \tag{S24}$$

obtaining the average of Eq. (S23) reduces to calculating the dimensionless quantity

$$\kappa_j = -\text{Tr}\left[\hat{D}_j(\xi_j^-)\left(\hat{b}_j + \hat{b}_j^\dagger\right)\hat{D}_j^\dagger(\xi_j^+)\rho_j^{(\text{th})}\right] \tag{S25}$$

$$= \left(\xi_j^+ + \xi_j^-\right) e^{-\frac{1}{2}\left(\xi_j^+ - \xi_j^-\right)^2 \coth\left(\frac{\omega_j}{2k_{\text{B}}T}\right)}$$

$$= 2\frac{\langle 1|\hat{V}_j|1\rangle}{\omega_j} e^{-2\frac{(w_j^z)^2}{\omega_j^2}\coth\left(\frac{\omega_j}{2k_{\text{B}}T}\right)}$$

$$= 2\frac{\langle 1|\hat{V}_j|1\rangle}{\omega_j}\left(1 + \mathscr{O}(B^2),\right)$$

which appears as a multiplicative rescaling factor for the off-diagonal couplings $\langle 1_\mp|\hat{W}_j|1_\pm\rangle$. Note that, when neglecting second and higher order terms in the magnetic field, κ_j does not show any dependence on temperature or on the magnetic field orientation via θ_1 and ϕ_1.

After thermal averaging, the effective electronic Hamiltonian for the lowest energy doublet becomes

$$\hat{H}_{\rm el} = {\rm Tr}_{\rm ph}\left[\hat{S}\hat{H}_{\rm eff}\hat{S}^{\dagger}\rho_{\rm ph}^{(\rm th)}\right] = E_1 + \delta E_1 + \left(2\sum_j \frac{\langle 1|\hat{V}_j|1\rangle}{\omega_j}w_j^x, 2\sum_j \frac{\langle 1|\hat{V}_j|1\rangle}{\omega_j}w_j^y, \frac{\Delta_1}{2} + 2\sum_j \frac{\langle 1|\hat{V}_j|1\rangle}{\omega_j}w_j^z\right)\cdot\begin{pmatrix}\sigma'_x\\ \sigma'_y\\ \sigma'_z\end{pmatrix} \tag{S26}$$

where the energy of the lowest doublet is shifted by

$$\delta E_1 = -\sum_j \frac{\langle 1|\hat{V}_j|1\rangle^2}{\omega_j} + \sum_j \frac{\omega_j}{e^{\omega_j/k_{\rm B}T}-1} \tag{S27}$$

due to the spin-phonon coupling and to the thermal phonon energy. Eq. (S26) thus represents a refined description of the lowest effective spin-1/2 doublet in the presence of spin-phonon coupling.

We can finally recast the Hamiltonian (S26) in terms of a g-matrix for an effective spin 1/2, similarly to what we did earlier in the case of no spin-phonon coupling. In order to do so, we first recall from Eq. (S6) and (S14) that the quantities Δ_1 and (w_j^x, w_j^y, w_j^z) appearing in Eq. (S26) depend on the magnetic field orientation via the states $|1_\pm\rangle$, and on both orientation and intensity via $\hat{H}_{\rm Zee}$. We can get rid of the first dependence by expressing the Zeeman eigenstates $|1_\pm\rangle$ in terms of the original crystal field eigenstates $|1\rangle$, $|\bar{1}\rangle$. For the spin-phonon coupling vector $\mathbf{w}_j$, we obtain

$$\mathbf{w}_j = \begin{pmatrix}\Re\langle 1_-|\hat{W}_j|1_+\rangle\\ \Im\langle 1_-|\hat{W}_j|1_+\rangle\\ \langle 1_+|\hat{W}_j|1_+\rangle\end{pmatrix} = \begin{pmatrix}\cos\theta_1\cos\phi_1 & \cos\theta_1\sin\phi_1 & -\sin\theta_1\\ -\sin\phi_1 & \cos\phi_1 & 0\\ \sin\theta_1\cos\phi_1 & \sin\theta_1\sin\phi_1 & \cos\theta_1\end{pmatrix}\begin{pmatrix}\Re\langle\bar{1}|\hat{W}_j|1\rangle\\ \Im\langle\bar{1}|\hat{W}_j|1\rangle\\ \langle 1|\hat{W}_j|1\rangle\end{pmatrix} = \mathbf{R}(\theta_1,\phi_1)\cdot\tilde{\mathbf{w}}_j. \tag{S28}$$

where $\mathbf{R}(\theta_1,\phi_1)$ is a rotation matrix. Similarly, the elctronic contribution Δ_1 transforms as

$$(0,0,\Delta_1) = \mathbf{j}_1\cdot\mathbf{R}(\theta_1,\phi_1)^T, = \mu_{\rm B}\mathbf{B}\cdot\mathbf{g}_{\rm el}^{(1)}\cdot\mathbf{R}(\theta_1,\phi_1)^T. \tag{S29}$$

The Pauli spin operators need to be changed accordingly to $\tilde{\boldsymbol{\sigma}} = \mathbf{R}(\theta_1,\phi_1)^T\cdot\boldsymbol{\sigma}'$. Lastly, we single out explicitly the magnetic field dependence of $\hat{W}_j$, defined in Eq. (S13), by introducing a three-component operator $\hat{\mathbf{K}}_j = (\hat{K}_j^x, \hat{K}_j^y, \hat{K}_j^z)$, such that

$$\begin{aligned}\hat{W}_j &= \mu_{\rm B}g_J\mathbf{B}\cdot\left(\hat{V}_j\hat{Q}_1\hat{\mathbf{J}} + \hat{\mathbf{J}}\hat{Q}_1\hat{V}_j\right)\\ &= \mu_{\rm B}g_J\mathbf{B}\cdot\hat{\mathbf{K}}_j.\end{aligned} \tag{S30}$$

Thus, the effective electronic Hamiltonian in Eq. (S26) can be finally rewritten as

$$\hat{H}_{\rm el} = E_1 + \delta E_1 + \mu_{\rm B}\mathbf{B}\cdot\left(\mathbf{g}_{\rm el}^{(1)} + \mathbf{g}_{\rm vib}\right)\cdot\tilde{\boldsymbol{\sigma}}/2 \tag{S31}$$

where $\mathbf{g}_{\rm el}^{(1)}$ is the electronic g-matrix defined in Eq. (S3), and

$$\mathbf{g}_{\rm vib} = 4g_J\sum_j \frac{\langle 1|\hat{V}_j|1\rangle}{\omega_j}\begin{pmatrix}\Re\langle\bar{1}|\hat{K}_j^x|1\rangle & \Im\langle\bar{1}|\hat{K}_j^x|1\rangle & \langle 1|\hat{K}_j^x|1\rangle\\ \Re\langle\bar{1}|\hat{K}_j^y|1\rangle & \Im\langle\bar{1}|\hat{K}_j^y|1\rangle & \langle 1|\hat{K}_j^y|1\rangle\\ \Re\langle\bar{1}|\hat{K}_j^z|1\rangle & \Im\langle\bar{1}|\hat{K}_j^z|1\rangle & \langle 1|\hat{K}_j^z|1\rangle\end{pmatrix} \tag{S32}$$

is a vibronic correction.

Note that this correction is non-perturbative in the spin-phonon coupling, despite only containing quadratic terms in $\hat{V}_j$ (recall that $\hat{\mathbf{K}}_j$ depends linearly on $\hat{V}_j$). The only approximations leading to Eq. (S31) are a linear perturbative expansion in the magnetic field $\mathbf{B}$ and neglecting quantum fluctuations of the off-diagonal spin-phonon coupling in the polaron frame, which is accounted for only via its thermal expectation value. This approximation relies on the fact that the off-diagonal couplings are much smaller than the diagonal spin-phonon coupling that is treated exactly by the polaron transformation (see Supplementary Note 3).

C. Landau-Zener probability

Let us consider a situation in which the magnetic field comprises a time-independent contribution arising from internal dipolar or hyperfine fields $\mathbf{B}_{\rm int}$ and a time dependent external field $\mathbf{B}_{\rm ext}(t)$. Let us fix the orientation of the external field and vary its magnitude at a constant rate, such that the field switches direction at $t = 0$. Under these circumstances, the Hamiltonian of Eq. (S31) becomes

$$\hat{H}_{\rm el}(t) = E_1 + \delta E_1 + \mu_{\rm B}\left(\mathbf{B}_{\rm int} + \frac{{\rm d}\mathbf{B}_{\rm ext}}{{\rm d}t}t\right)\cdot\mathbf{g}\cdot\frac{\tilde{\boldsymbol{\sigma}}}{2}, \tag{S33}$$

where $\mathbf{g} = \mathbf{g}_{\mathrm{el}}^{(1)} + \mathbf{g}_{\mathrm{vib}}$. Neglecting the constant energy shift and introducing the vectors

$$\boldsymbol{\Delta} = \mu_{\mathrm{B}} \mathbf{B}_{\mathrm{int}} \cdot \mathbf{g}, \tag{S34}$$
$$\mathbf{v} = \mu_{\mathrm{B}} \mathrm{d}\mathbf{B}_{\mathrm{ext}}/\mathrm{d}t \cdot \mathbf{g}, \tag{S35}$$

the Hamiltonian then becomes

$$\hat{H}_{\mathrm{el}}(t) = \frac{\boldsymbol{\Delta}}{2} \cdot \tilde{\boldsymbol{\sigma}} + \frac{\mathbf{v}t}{2} \cdot \tilde{\boldsymbol{\sigma}} = \frac{\boldsymbol{\Delta}_{\perp}}{2} \cdot \tilde{\boldsymbol{\sigma}} + \frac{\mathbf{v}t + \boldsymbol{\Delta}_{\parallel}}{2} \cdot \tilde{\boldsymbol{\sigma}}. \tag{S36}$$

In the second equality, we have split the vector $\boldsymbol{\Delta} = \boldsymbol{\Delta}_{\perp} + \boldsymbol{\Delta}_{\parallel}$ into a perpendicular and a parallel component to $\mathbf{v}$. Choosing an appropriate reference frame, we can write

$$\hat{H}_{\mathrm{el}}(t') = \frac{\Delta_{\perp}}{2} \tilde{\sigma}_x + \frac{vt'}{2} \tilde{\sigma}_z, \tag{S37}$$

in terms of the new time variable $t' = t + \Delta_{\parallel}/v$. Assuming that the spin is initialised in its ground state at $t' \to -\infty$, the probability of observing a spin flip at $t' \to +\infty$ is given by the Landau-Zener formula [3–8]

$$P_{\mathrm{LZ}} = 1 - \exp\left(-\frac{\pi \Delta_{\perp}^2}{2v}\right). \tag{S38}$$

We remark that tunnelling is only made possible by the presence of $\Delta_{\perp}$, which stems from internal fields that have a perpendicular component to the externally applied field. We also observe that a perfectly axial system would not exhibit tunnelling behaviour, since in that case the direction of $\mathbf{B} \cdot \mathbf{g}$ would always point along the easy axis (i.e. along the only eigenvector of $\mathbf{g}$ with a non-vanishing eigenvalue), and therefore $\mathbf{v}$ and $\boldsymbol{\Delta}$ would always be parallel. Thus, deviations from axiality and the presence of transverse fields are both required for QTM to occur.

Supplementary Note 3. DISTRIBUTION OF SPIN-PHONON COUPLING VECTORS

The effective polaron Hamiltonian presented in the main text and derived in the previous section provides a good description of the ground doublet only if the spin-phonon coupling operators are approximately diagonal in the electronic eigenbasis. This is equivalent to requiring that the components of the vectors $\mathbf{w}_j$ defined in Eq. (S14) satisfy

$$|w_j^x|, |w_j^y| \ll |w_j^z|. \tag{S39}$$

Thus, a value of $\|\mathbf{w}_j^{\perp}\| = \sqrt{(w_j^x)^2 + (w_j^y)^2}$ much smaller than $\|\mathbf{w}_j\|$ ensures that a polaron model is well justified. However, we stress that, even when this condition is not met, the polaron Hamiltonian of Eq. (S26) still accounts for the transverse spin-phonon couplings w_j^x and w_j^y in an effective way by considering their thermal average.

Supplementary Fig. 2 shows the values $\|\mathbf{w}_j^{\perp}\|/\|\mathbf{w}_j\|$, which determine the validity of the polaron approximation, for all modes $\{\mathbf{w}_j, j = 1, \ldots, M\}$ (where M is the number of vibrational modes) under the effect of a magnetic field applied in the hard plane, $\mathbf{B} = (1, 0, 0)$, or along the easy axis, $\mathbf{B} = (0, 0, 1)$. The polaron approximation is well justified by the observation that, for most vibrational modes, $\|\mathbf{w}_j^{\perp}\|/\|\mathbf{w}_j\|$ is below 0.01 for $[\mathrm{Dy(Cp^{ttt})_2}]^+$ and below 0.1 for [Dy(bbpen)Br].

This observation is confirmed by comparing the variance of the set of vectors $\{\mathbf{w}_j\}$ in the xy-plane, $\sigma_x^2 + \sigma_y^2$, to the total variance, $\sigma^2 = \sigma_x^2 + \sigma_y^2 + \sigma_z^2$, where

$$\sigma_\alpha^2 = \mathrm{var}(w_j^\alpha) = \frac{1}{M} \sum_{j=1}^{M} \left(w_j^\alpha - \mu_\alpha \right)^2, \tag{S40}$$

with $\alpha = x, y, z$ and $\mu_\alpha = \frac{1}{M} \sum_{j=1}^{M} w_j^\alpha$. For $[\mathrm{Dy(Cp^{ttt})_2}]^+$, the variance in xy-plane only accounts for around 10^{-6} of the total variance, whereas for [Dy(bbpen)Br] the fraction goes up to 10^{-3}. Therefore, we conclude that the approach followed in Supplementary Note 2 is fully justified.

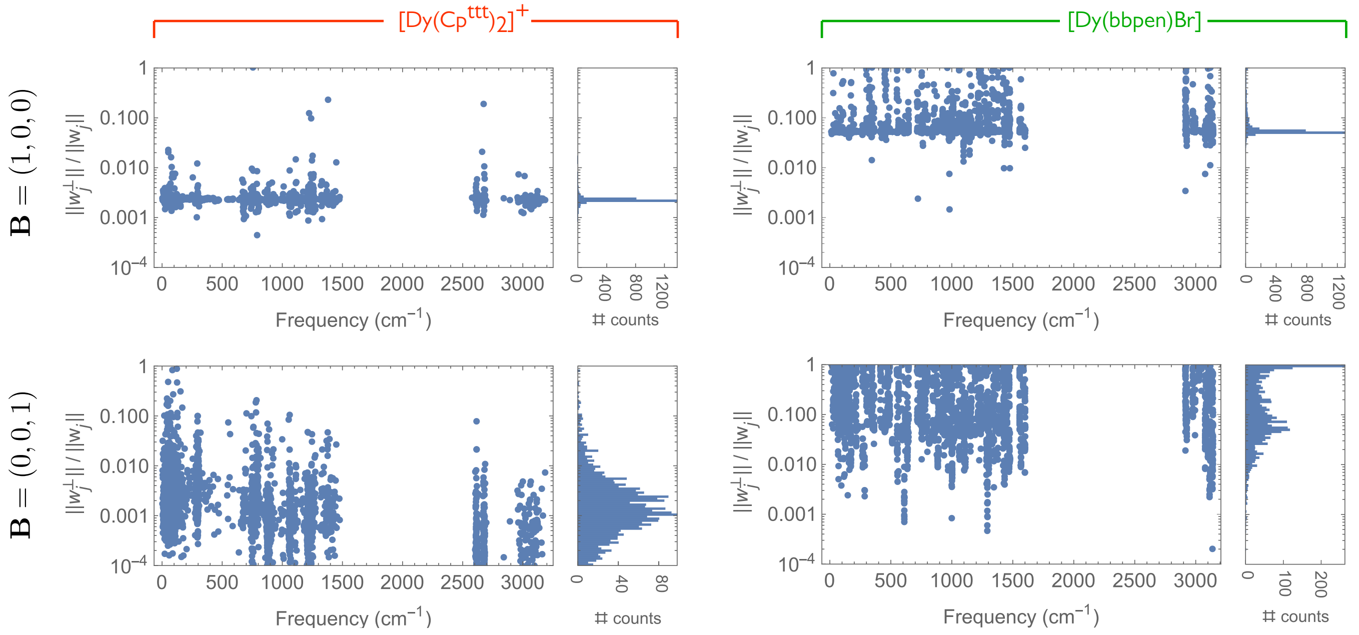

Supplementary Figure 2. **Distribution of transverse spin-phonon coupling strength $\|\mathbf{w}_j^{\perp}\|/\|\mathbf{w}_j\|$.** The transverse spin-phonon coupling vector $\mathbf{w}_j^{\perp}$ is the projection onto the hard plane of the spin-phonon coupling vector $\mathbf{w}_j$. Left: $[\mathrm{Dy(Cp^{ttt})_2}]^+$; right: [Dy(bbpen)Br]; top: magnetic field $\mathbf{B}$ oriented along x (hard plane); bottom: magnetic field $\mathbf{B}$ oriented along z (easy axis). The field magnitude is fixed to 1 T.

Supplementary Note 4. GROUND ZEEMAN SPLITTING FOR [Dy(bbpen)Br]

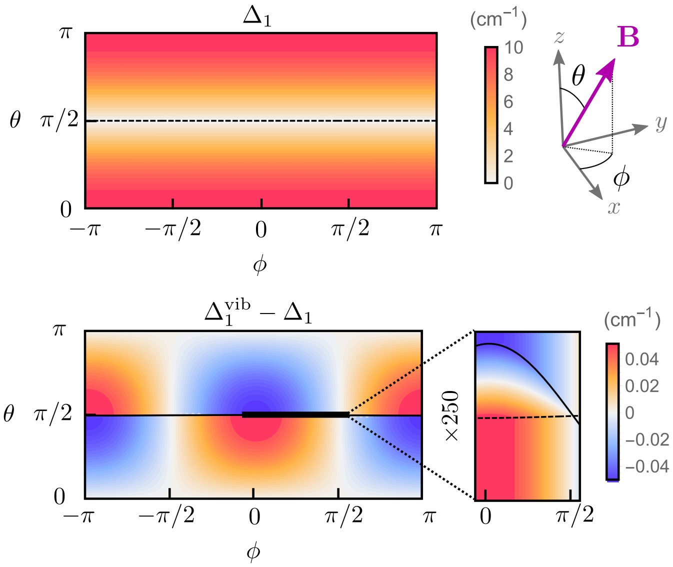

Supplementary Figure 3. **Zeeman splitting of the ground Kramers doublet in [Dy(bbpen)Br]. a**, Electronic ground doublet splitting (Δ_1, top) and vibronic correction ($\Delta_1^{\rm vib} - \Delta_1$, bottom) as a function of the orientation of the magnetic field, parametrised in terms of polar and azimuthal angles θ and ϕ. The polar angle θ is measured with respect to the axis joining the two oxygen atoms, corresponding approximately to the easy axis. The dashed (solid) line corresponds to the electronic (vibronic) hard plane. The magnitude of the magnetic field is fixed to 1 T.

Supplementary Note 5. ESTIMATE OF THE INTERNAL FIELDS

A. Dipolar fields

In this section we provide an estimate of the internal fields B_{int} in a disordered ensemble of SMMs. When a SMM with strongly axial magnetic anisotropy is placed in a strong external magnetic field $\mathbf{B}_{\text{ext}}$, it gains a non-zero magnetic dipole moment along its easy axis. Once the external field is removed, the SMM partially retains its magnetisation $\boldsymbol{\mu} = \mu\hat{\boldsymbol{\mu}}$, which produces a microscopic dipolar field

$$\mathbf{B}_{\text{dip}}(\mathbf{r}) = \frac{\mu_0\mu}{4\pi r^3}\left[3\hat{\mathbf{r}}(\hat{\boldsymbol{\mu}}\cdot\hat{\mathbf{r}}) - \hat{\boldsymbol{\mu}}\right] \tag{S41}$$

at a point $\mathbf{r} = r\hat{\mathbf{r}}$ in space. This field can then cause a tunnelling gap to open in neighboring SMMs, depending on their relative distance and orientation.

Knowing the spatial distribution and orientation of an ensemble of SMMs, either amorphous or crystalline, we can estimate the internal field experienced by a randomly selected SMM in the ensemble due to all other members of the ensemble.

Frozen solution — In the case of a $[\text{Dy}(\text{Cp}^{\text{ttt}})_2]^+$ frozen solution, we consider a uniform distribution of randomly oriented SMMs in a sphere of radius R around a central SMM placed at $\mathbf{r} = 0$. We choose a 170 mM SMM concentration to mimic typical experimental conditions [9]. The ground magnetic moment of the Dy centres can be determined by reading the saturation value of the magnetisation M_{sat} of a frozen solution sample of known volume V and concentration c, containing $N = cV$ magnetic centres. Using data from ref. [9], we obtain an average magnetic moment per molecule

$$\langle\mu_\parallel\rangle = \frac{M}{N} \approx 4.07\mu_{\text{B}} \tag{S42}$$

along the direction of the external field $\mathbf{B}_{\text{ext}}$, where $\langle\cdot\rangle$ denotes the average over the ensemble of SMMs. Since the orientation of SMMs in a frozen solution is random, the component of the magnetisation $\boldsymbol{\mu}$ perpendicular to the applied field averages to zero, i.e. $\langle\mu_\perp\rangle = 0$. However, it still contributes to the formation of the microscopic dipolar field (S41), which depends on $\boldsymbol{\mu} = \boldsymbol{\mu}_\parallel + \boldsymbol{\mu}_\perp$. Since the sample consists of many randomly oriented SMMs, the average magnetisation in Eq. (S42) can also be expressed in terms of $\mu = |\boldsymbol{\mu}|$ via the orientational average

$$\langle\mu_\parallel\rangle = \int_0^{\pi/2} \mathrm{d}\theta\ \sin\theta\ \mu_\parallel(\theta) = \frac{\mu}{2}, \tag{S43}$$

where $\mu_\parallel(\theta) = \mu\cos\theta$ is the component of the magnetisation of a SMM along the direction of the external field $\mathbf{B}_{\text{ext}}$. Thus, the magnetic moment responsible for the microscopic dipolar field is twice as big as the measured value (S42). We enforce a minimum distance of 10 Å between dipoles, corresponding to approximately twice the RMS distance of ligand atoms from Dy. Although the dipoles are randomly oriented, the orientation of the dipole is chosen such that the z-component is always positive to simulate the presence of an external field $\mathbf{B}_{\text{ext}}$ along z. We repeat this process 10,000 times in order to sample the full distribution of fields and spin-flip probabilities. The resulting dipolar field is randomly oriented and has an average magnitude of 5.54 mT, as shown in Supplementary Fig. 4a. The corresponding spin-flip probabilities are calculated via Landau-Zener theory (Supplementary Note 2) and are shown in Fig. 3b. We checked convergence with respect to the solvent sphere radius R and see no significant changes for average number of dipoles ranging from 125 to 1000 (Table 1).

$\langle N\rangle$	R (Å)	$\langle B_{\text{dip}}\rangle$ (mT)	SD	$\langle P_{\text{LZ}}^{(\text{el})}\rangle$	SD	$\langle P_{\text{LZ}}^{(\text{vib})}\rangle$	SD
125	66	5.49	3.22	0.0104	0.0147	0.244	0.234
250	84	5.47	3.24	0.0105	0.0147	0.245	0.235
500	105	5.48	3.23	0.0105	0.0147	0.247	0.234
1000	133	5.54	3.25	0.0107	0.0148	0.250	0.238

Supplementary Table 1. **Monte Carlo dipolar field and spin-flip probability of different sized solvent balls.** Average values of dipolar field magnitude $\langle B_{\text{dip}}\rangle$, electronic and vibronic spin-flip probabilities $\langle P_{\text{LZ}}^{(\text{el})}\rangle$, $\langle P_{\text{LZ}}^{(\text{vib})}\rangle$, and their standard deviations (SD) are reported side by side. The average number of magnetic dipoles included in the calculation is denoted by $\langle N\rangle$, corresponding to a sphere of radius R, assuming magnetic dipoles have a concentration of 170 mM.

Molecular crystal — In the case of [Dy(bbpen)Br], the spatial distribution of dipoles is fully determined by the crystal structure. In order to account for polycrystalline samples, we sample random orientations of the magnetising field $\mathbf{B}_{\text{ext}}$ with respect to the crystal orientation. Another source of randomness in this molecular crystal comes from diamagnetic dilution of Dy in Y. This is mimicked by setting to zero the dipole moments at the Dy lattice positions with 95% probability [10]. The ground magnetic moment was fixed to $10\mu_{\text{B}}$, owing to the observation of fully saturated magnetisation at 1 T [10]. We consider the field

produced by all magnetic dipoles within a sphere of radius $R = 100$ Å centred on a Dy atom and repeat this process 10,000 times in order to sample the full distribution of fields shown in Supplementary Fig. 4b. While the average magnitude of the dipolar field is similar to the one obtained for the frozen solution, its orientation is not isotropic. The component the direction joining two Dy centres belonging to the same unit cell (z in Supplementary Fig. 4b) averages to 2.93 mT. Since the principal anisotropy axis of Dy forms a 23° angle with respect to that direction, this field results in an average 1.15 mT transverse component. The presence of this no-vanishing transverse field explains the much higher QTM probabilities obtained in this case.

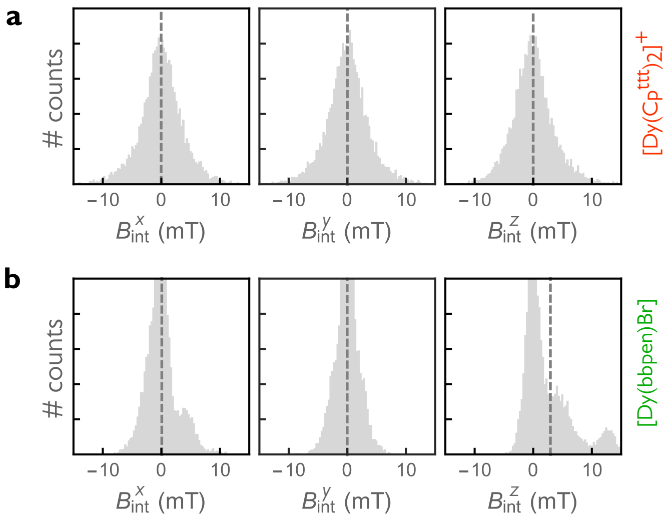

Supplementary Figure 4. **Dipolar field distribution.** Distributions of the cartesian components of $\mathbf{B}_{\rm int} = (B^x_{\rm int}, B^y_{\rm int}, B^z_{\rm int})^T$. Average values are indicated by vertical dashed lines. **a**, $[{\rm Dy(Cp^{ttt})_2}]^+$ in dichloromethane solvent ball; **b**, [Dy(bbpen)Br] molecular crystal.

B. Hyperfine coupling

Another possible source of microscopic magnetic fields are nuclear spins. Among the different isotopes of dysprosium, only ^{161}Dy and ^{163}Dy have non-zero nuclear spin ($I = 5/2$), making up for approximately 44 % of naturally occurring dysprosium. The nucear spin degrees of freedom are described by the Hamiltonian

$$\hat{H}_{\rm nuc} = \hat{H}_{\rm Q} + \hat{H}_{\rm HF} = \hat{\mathbf{I}} \cdot \mathbf{P} \cdot \hat{\mathbf{I}} + \hat{\mathbf{I}} \cdot \mathbf{A} \cdot \hat{\mathbf{J}}, \tag{S44}$$

where the first term is the quadrupole Hamiltonian $\hat{H}_{\rm Q} = \hat{\mathbf{I}} \cdot \mathbf{P} \cdot \hat{\mathbf{I}}$, accounting for the zero-field splitting of the nuclear spin states, and the second term $\hat{H}_{\rm HF} = \hat{\mathbf{I}} \cdot \mathbf{A} \cdot \hat{\mathbf{J}}$ accounts for the hyperfine coupling between nuclear spin $\hat{\mathbf{I}}$ and electronic angular momentum $\hat{\mathbf{J}}$ operators. In analogy with the electronic Zeeman Hamiltonian $\hat{H}_{\rm Zee} = \mu_{\rm B} g_J \mathbf{B} \cdot \hat{\mathbf{J}}$, we define the effective nuclear magnetic field operator

$$\mu_{\rm B} g_J \hat{\mathbf{B}}_{\rm nuc} = \mathbf{A}^T \cdot \hat{\mathbf{I}}, \tag{S45}$$

so that the hyperfine coupling Hamiltonian takes the form of a Zeeman interaction $\hat{H}_{\rm HF} = \mu_{\rm B} g_J \hat{\mathbf{B}}^\dagger_{\rm nuc} \cdot \hat{\mathbf{J}}$. If we consider the nuclear spin to be in a thermal state at temperature T with respect to the quadrupole Hamiltonian $\hat{H}_{\rm Q}$, the resulting expectation value of the nuclear magnetic field vanishes, since the nuclear spin is completely unpolarised. However, the external field $\mathbf{B}_{\rm ext}$ will tend to polarise the nuclear spin via the nuclear Zeeman Hamiltonian

$$\hat{H}_{\rm nuc,\, Zee} = \mu_{\rm N} g_I\, \mathbf{B}_{\rm ext} \cdot \hat{\mathbf{I}}, \tag{S46}$$

where $\mu_{\rm N}$ is the nuclear magneton and g_I is the nuclear g-factor of a Dy nucleus. In this case, the nuclear spin is described by the thermal state

$$\rho^{\rm (th)}_{\rm nuc} = \frac{e^{-(\hat{H}_{\rm Q} + \hat{H}_{\rm nuc,\, Zee})/k_{\rm B} T}}{{\rm Tr}\left[e^{-(\hat{H}_{\rm Q} + \hat{H}_{\rm nuc,\, Zee})/k_{\rm B} T} \right]} \tag{S47}$$

and the effective nuclear magnetic field can be calculated as

$$\mathbf{B}_{\text{nuc}} = \text{Tr}\left[\hat{\mathbf{B}}_{\text{nuc}}\rho_{\text{nuc}}^{(\text{th})}\right] . \tag{S48}$$

To the best of our knowledge, quadrupole and hyperfine coupling tensors for Dy in $[\text{Dy}(\text{Cp}^{\text{ttt}})_2]^+$ and [Dy(bbpen)Br] have not been reported in the literature. However, ab initio calculations of hyperfine coupling tensors have been performed on DyPc_2 [11]. Although the dysprosium atom in DyPc_2 and $[\text{Dy}(\text{Cp}^{\text{ttt}})_2]^+$ interacts with different ligands, the crystal field is qualitatively similar for these two complexes, therefore we expect the nuclear spin Hamiltonian to be sufficiently close to the one for $[\text{Dy}(\text{Cp}^{\text{ttt}})_2]^+$, at least for the purpose of obtaining an approximate estimate. Using the quadrupolar and hyperfine tensors determined for DyPc_2 [11] and the nuclear g-factors measured for ^{161}Dy and ^{163}Dy [12], we can compute $B_{\text{nuc}} = |\mathbf{B}_{\text{nuc}}|$ from Eq. (S48) for different orientations of the external magnetic field. As shown in Supplementary Table 2, the effective nuclear magnetic fields at $T = 2$ K are at least one order of magnitude smaller than the dipolar fields calculated in the previous section, regardless of the orientation of the external field.

	^{161}Dy	^{163}Dy
$\mathbf{B}_{\text{ext}} \parallel \hat{\mathbf{x}}$	2.82×10^{-8} T	5.34×10^{-8} T
$\mathbf{B}_{\text{ext}} \parallel \hat{\mathbf{y}}$	1.77×10^{-8} T	3.38×10^{-8} T
$\mathbf{B}_{\text{ext}} \parallel \hat{\mathbf{z}}$	5.51×10^{-5} T	1.08×10^{-4} T

Supplementary Table 2. **Effective nuclear magnetic field.** The effective field due to hyperfine coupling is calculated using Eq. (S48), assuming the nuclear spin to be in a thermal state at temperature $T = 2$ K. Different rows correspond to different orientations of the external magnetic field $\mathbf{B}_{\text{ext}}$, chosen to lie along the three cartesian unit vectors $\hat{\mathbf{x}}$, $\hat{\mathbf{y}}$, $\hat{\mathbf{z}}$. Columns correspond to the two naturally occurring isotopes of Dy.

Supplementary Note 6. RELATION BETWEEN SINGLE-MODE AXIALITY AND SPIN-FLIP PROBABILITY

In the following we show that the correlation between single-mode spin-flip probability $\langle P_j \rangle$ and single mode axiality A_j presented in Fig. 4 in the main text can be rationalised in terms of a simple toy model.

Let us work in the reference frame where the electronic g-matrix is diagonal. For a system with strong easy-axis character, this can be approximated as

$$\mathbf{g}_{\text{el}} \propto \begin{pmatrix} \lambda & & \\ & \lambda & \\ & & 1 \end{pmatrix} \quad \text{with} \quad \lambda \ll 1. \tag{S49}$$

We choose the vibronic correction to the g-matrix to have easy-axis anisotropy as well and we only consider its largest g-value η, which is also much less than one. This approximation is justified by inspection of the g-matrices $\mathbf{g}_{\text{vib}}$ obtained numerically. The direction of the anisotropy axis corresponding to η is determined by θ, the tilt angle away from the electronic hard plane, as sketched in Supplementary Fig. 5a. Thus,

$$\mathbf{g}_{\text{vib}} \propto \eta \begin{pmatrix} 0 & 0 & 0 \\ 0 & \cos^2\theta & \sin\theta\cos\theta \\ 0 & \sin\theta\cos\theta & \sin^2\theta \end{pmatrix}. \tag{S50}$$

Assuming an external field sweep along z and an internal field along y, we can calculate axiality and spin-flip probability corresponding to both the electronic g-matrix $\mathbf{g}_{\text{el}}$ and the vibronic one $\mathbf{g}_{\text{el}} + \mathbf{g}_{\text{vib}}$. If no spin-phonon coupling is present ($\eta = 0$), we obtain

$$A_{\text{el}} = \tfrac{1-\lambda}{1+2\lambda}, \tag{S51}$$

$$P_{\text{el}} = 1 - e^{-C\lambda^2}, \tag{S52}$$

where C is a positive constant determined by sweep rate, internal field and absolute value of the largest electronic g-value. Assuming weak spin-phonon coupling for simplicity, the vibronic analogue of these quantities can be expanded in powers of η as

$$A_{\text{vib}} = A_{\text{el}} + \alpha(\theta,\lambda)\eta + O(\eta^2), \tag{S53}$$

$$P_{\text{vib}} = P_{\text{el}} + \beta(\theta,\lambda)\eta + O(\eta^2), \tag{S54}$$

where

$$\alpha(\theta,\lambda) = -\tfrac{3}{4}\tfrac{1-2\lambda+(1+2\lambda)\cos 2\theta}{(1+2\lambda)^2} \tag{S55}$$

$$\beta(\theta,\lambda) = \tfrac{1}{2}C\lambda e^{-C\lambda^2}\left(2-\lambda+(2+\lambda)\cos 2\theta\right). \tag{S56}$$

If the two coefficients α and β have opposite signs ($\alpha\beta < 0$), axiality A_{vib} and spin-flip probability P_{vib} become anti-correlated: switching on the spin-phonon coupling ($\eta \neq 0$) will increase one at the expenses of the other. In order to satisfy the condition $\alpha\beta < 0$, the parameters θ and λ need to be chosen such that

$$\cos 2\theta < -\frac{1-\lambda/2}{1+\lambda/2} \quad \text{or} \quad \cos 2\theta > -\frac{1-2\lambda}{1+2\lambda}. \tag{S57}$$

Note that this result only depends on the relative orientation of electronic and vibrational easy axis (θ) and degree of electronic axiality (determined by λ). For a given value of λ, Eq. (S57) is satisfied for all angles θ, except the ones falling in the grey shaded region in Supplementary Fig. 5b. Assuming a uniformly distributed angle θ across several vibrational modes, spin-phonon coupling will lead to negative correlation between A_{vib} and P_{vib}. The window of values for θ that does not lead to this behaviour becomes increasingly smaller upon increasing the axiality of the electronic g-matrix, i.e. decreasing λ.

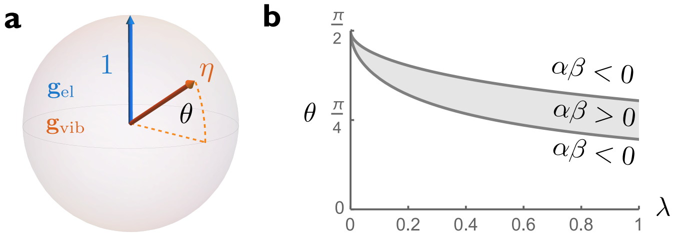

Supplementary Figure 5. **Toy model for vibronic axiality. a**, Main anisotropy axis for the electronic (blue) and vibrational (orange) g-matrices $\mathbf{g}_{el}$ and $\mathbf{g}_{vib}$, with corresponding g-values 1 and η. The angle θ is defined as the angle between the vibrational easy axis and the electronic hard plane. **b**, Values of angle θ and electronic hard-plane g-value λ that lead to the observed anti-correlation between axiality and spin-flip probability. The shaded area between the two gray lines corresponds to the domain where Eq. (S57) is not satisfied. α and β are the linear coefficients of the Taylor expansion of vibronic axiality and spin-flip probability with respect to η, defined in Eq. (S55) and (S56).

SUPPLEMENTARY REFERENCES

[1] J. G. C. Kragskow, A. Mattioni, J. K. Staab, D. Reta, J. M. Skelton, and N. F. Chilton, *Spin-phonon coupling and magnetic relaxation in single-molecule magnets,* Chem. Soc. Rev. **52**, 4567 (2023).
[2] L. F. Chibotaru, A. Ceulemans, and H. Bolvin, *Unique definition of the Zeeman-splitting g tensor of a Kramers doublet,* Phys. Rev. Lett. **101**, 033003 (2008).
[3] L. D. Landau, *Zur Theorie der Energieübertragung,* Phyz. Z. Sowjetunion **1**, 88 (1932).
[4] L. D. Landau, *Zur Theorie der Energieübertragung II,* Phyz. Z. Sowjetunion **2**, 46 (1932).
[5] C. Zener and R. H. Fowler, *Non-adiabatic crossing of energy levels,* Proceedings of the Royal Society of London. Series A **137**, 696 (1932).
[6] E. C. G. Stückelberg, *Theorie der unelastischen Stösse zwischen Atomen,* Helv. Phys. Acta **5**, 369 (1932).
[7] E. Majorana, *Atomi orientati in campo magnetico variabile,* Il Nuovo Cimento **9**, 43 (1932).
[8] O. V. Ivakhnenko, S. N. Shevchenko, and F. Nori, *Nonadiabatic Landau-Zener-Stückelberg-Majorana transitions, dynamics, and interference,* Physics Reports **995**, 1 (2023).
[9] C. A. P. Goodwin, F. Ortu, D. Reta, N. F. Chilton, and D. P. Mills, *Molecular magnetic hysteresis at 60 kelvin in dysprosocenium,* Nature **548**, 439 (2017).
[10] J. Liu, Y.-C. Chen, J.-L. Liu, V. Vieru, L. Ungur, J.-H. Jia, L. F. Chibotaru, Y. Lan, W. Wernsdorfer, S. Gao, X.-M. Chen, and M.-L. Tong, *A stable pentagonal bipyramidal Dy(III) single-ion magnet with a record magnetization reversal barrier over 1000 K,* Journal of the American Chemical Society **138**, 5441 (2016).
[11] A. L. Wysocki and K. Park, *Hyperfine and quadrupole interactions for Dy isotopes in $DyPc_2$ molecules,* Journal of Physics: Condensed Matter **32**, 274002 (2020).
[12] J. Ferch, W. Dankwort, and H. Gebauer, *Hyperfine structure investigations in DyI with the atomic beam magnetic resonance method,* Physics Letters A **49**, 287 (1974).
